# Outcomes of a brief coping skills group intervention for adults with severe postconcussion symptoms

**DOI:** 10.2217/cnc-2019-0011

**Published:** 2019-11-21

**Authors:** Jordan I Ali, Patricia Mahoney, Derry Dance, Noah D Silverberg

**Affiliations:** 1 Department of Psychology, University of Victoria, Victoria, BC, V8W 2Y2, Canada; 2 Acquired Brain Injury Program, GF Strong Rehabilitation Centre, Vancouver, BC, V5Z 2G9, Canada; 3 Division of Physical Medicine & Rehabilitation, University of British Columbia, Vancouver, BC, V5Z 2G9, Canada; 4 Rehabilitation Research Program, Vancouver Coastal Health Research Institute, Vancouver, BC, V5Z 2G9, Canada

**Keywords:** concussion, mild TBI, postconcussion syndrome, rehabilitation, treatment

## Abstract

**Aim:**

The aim of this study was to evaluate a brief psychologically informed coping skills group intervention for adults with severe prolonged symptoms following mild traumatic brain injury (mTBI).

**Methodology & results:**

Patients attended an education session about mTBI; 22 patients completed an additional coping skills group intervention, 16 declined/stopped the intervention early and 19 were not offered the intervention. At follow-up, patients who completed the intervention reported a similar degree of symptom improvement and disability as those who did not complete the intervention. The majority of patients who completed the intervention were satisfied with it and perceived it to be credible.

**Conclusion:**

The coping skills intervention was not associated with measurable clinical benefit. Recommendations for improving psychological interventions for mTBI are discussed.

Mild traumatic brain injury (mTBI) symptoms are generally expected to remit within 3 months; however, a substantial minority of patients who sustain an mTBI will experience symptoms that persist over the longer term [1]. Psychosocial factors, such as anxiety and maladaptive illness beliefs, are increasingly understood to be important perpetuating factors of chronic symptoms [2,3]. In light of this, early psychologically informed intervention is considered a promising approach to prevent prolonged symptoms after mTBI. Early cognitive behavioral therapy (CBT) may be an effective complement to education and reassurance [4,5,6], but the available evidence is limited and access to an individual therapist may be a barrier to implementation.

The current study describes a secondary analysis of a clinical quality improvement database compiled for the purposes of evaluating a brief CBT-informed coping skills group intervention for patients with severe prolonged symptoms after mTBI. Patients with severe, prolonged symptoms were targeted because they had the greatest need and potential to benefit from CBT and because CBT may be associated with a negligible group-level treatment effect when delivered to an unselected cohort [7]. The objectives of our secondary analysis were to provide preliminary evidence regarding the impact of the intervention on postconcussion symptom severity and self-reported disability. Further, we examined patient satisfaction with and perceived credibility of the coping skills intervention.

## Methods

As a secondary analysis of a de-identified clinical database, the present study was exempt from University of British Columbia research ethics board approval but was conducted in accordance with the Declaration of Helsinki.

Eligible patients were adults (aged 19+ years) referred by a physician to a specialty outpatient program, the Early Response Concussion Service at the GF Strong Rehabilitation Centre (BC, Canada). Patients first attended a 2-hour group education session about recovery from mTBI and symptom self-management strategies, led by an occupational therapist. During the session, patients completed questionnaires regarding their postconcussion symptoms (Rivermead Post Concussion Symptoms Questionnaire; RPQ [8]) and emotional health (Hospital Anxiety and Depression Scale; HADS [9]) until an interim analysis suggested that administering both measures was redundant for screening, as highly elevated RPQ scores (>40) rarely occurred without correspondingly elevated HADS scores (>7; frequency = 0/64, 0%).

**Figure 1.**
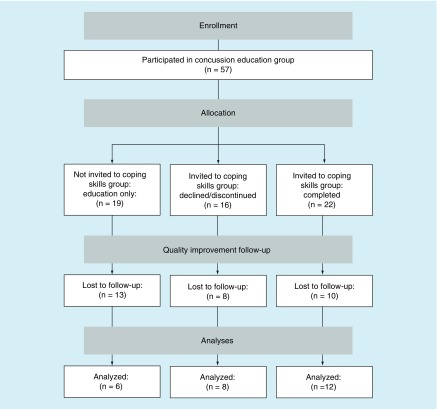
Patient flow.

Education session attendees who were less than 3 months post-injury and met criteria for high symptom burden were considered eligible for additional intervention – the new coping skills group as described below. High symptom burden was defined as either RPQ > 40 or both RPQ > 24 and HADS anxiety or depression subscale >7 [9]. Note that RPQ >24 falls above the 90th percentile for clinical norms [10]. Some otherwise eligible patients were not offered the group because their program entry coincided with therapist unavailability (i.e., low staffing), creating a natural control group of patients who did not receive the intervention of interest. A clinician blinded to intervention condition attempted to contact patients for quality improvement purposes at a minimum of 3 months after their program exit. Available patients completed self-report measures by telephone. Figure 1 illustrates the flow of patients through the study.

### Coping skills group intervention

Weekly 90-min sessions were co-delivered by an occupational therapist (P Mahoney) and a neuropsychologist (ND Silverberg) over 3 weeks, in cohorts of four to eight patients. Session 1 focused on developing a gradual return to activity plan and initiating its implementation. Session 2 oriented patients to the CBT model and how to apply cognitive reframing to common maladaptive illness beliefs after mTBI. In Session 3, participants were introduced to the overlap between symptoms of stress and symptoms of mTBI and were instructed on how to use relaxation strategies, including diaphragmatic breathing and progressive muscle relaxation. Patients completed homework assignments between sessions to assist with internalizing and generalizing these coping skills. A treatment manual with preprinted homework sheets was used to promote treatment fidelity. The full treatment manual is available from the authors upon request.

### Measures

Rivermead Post Concussion Symptoms Questionnaire (RPQ) [8]. The RPQ is a reliable and frequently used measure of postconcussion symptom burden. Respondents rate their experience of 16 common postconcussion symptoms on a scale from 0 (‘not experienced at all’) to 4 (‘a severe problem’). An RPQ score of 1 (‘no more of a problem’ following mTBI) denotes that a given symptom was present prior to the mTBI and continues to be present at the same (pre-injury) severity level. To determine the severity of symptoms with onset or aggravation following mTBI specifically, a total symptom burden score was derived by summing all item scores >1.

World Health Organization Disability Assessment Schedule 2.0 12-item interview version (WHODAS) [11]. The WHODAS is a disease-nonspecific measure of six International Classification of Disability, Functioning and Health domains, including cognition, mobility, self-care, interpersonal functioning, life activities and participation. Respondents rate their level of disability in each domain on a scale from 0 (‘None’) to 4 (‘Extreme/Cannot do’). The WHODAS is a reliable measure of disability after mTBI [11].

Patients who completed the coping skills group were also asked to rate their satisfaction with the coping skills intervention (‘How satisfied were you with the coping skills group?’) as well as ‘How likely is it that (they) would recommend the coping skills group to a friend/family member in a similar situation?’ on a scale from 1 (‘not at all’) to 5 (‘extremely’). The latter question was adapted from a legacy instrument for measuring perceived credibility of psychotherapies [12].

### Analyses

Demographic factors were compared across patients who completed the coping skills intervention, declined or discontinued the coping skills intervention (attended <2 of 3 sessions) or were not offered the coping skills intervention due to staffing limitations. Continuous variables were calculated using multivariate Analysis of Variance with group (three levels) as the independent variable and categorical variables were calculated using chi-squared tests. Demographic comparisons between patients available and unavailable for follow-up were conducted with independent samples t-tests. Pre-post symptom change (RPQ) and disability ratings (WHODAS) were calculated for patients who could be reached for follow-up. Total pre-post symptom change (RPQ) was calculated with repeated samples t-test. Group comparisons of disability (WHODAS) and rate of symptom change (RPQ) were conducted via univariate Analysis of Variance with group (3 levels) as the independent variable. A p-value <0.05 was considered statistically significant for all analyses.

## Results

There were no significant differences between intervention conditions with regard to sex, age or time since injury. Patients who declined or discontinued the coping skills intervention had significantly less involvement in litigation/compensation claims versus those who completed the coping skills intervention or were not offered the coping skills intervention (χ^2^[2, *N* = 57] = 8.56), p = 0.01 (Table 1). A total of 25 patients completed follow-up, one patient partially completed follow-up (excluding satisfaction and credibility items) and 31 patients either declined or were unavailable for follow-up after three attempts to contact them (Figure 1). There was no significant difference in intervention condition representation at follow-up. There were no significant differences in baseline characteristics between patients available and unavailable for follow-up, except that patients who completed follow-up endorsed significantly lower symptom burden (RPQ) at intake than patients who did not (t[50] = 2.25, p = 0.03).

**Table 1.** Demographics and outcome measures by group.

Total N = 57	Coping skills: not offered Total n = 19 Follow-up n = 6	Coping skills: declined/discontinued Total n = 16 Follow-up n = 8	Coping skills: completed Total n = 22 Follow-up n = 12	Follow-up: completed n = 26	Follow-up: declined/unavailable n = 31
Female n (%)	16 (84.2)	11 (68.8)	15 (68.2)	17 (65.4)	25 (80.6)
Age M (SD)	35.0 (11.0)	36.4 (14.0)	38.3 (10.5)	36.3 (11.8)	36.8 (11.5)
Personal injury litigation n (%)	10 (52.6)	1 (6.3)^†^	8 (36.4)	6 (23.1)	13 (41.9)
Motor vehicle accident n (%)	12 (63.2)	4 (25.0)	14 (63.6)	17 (65.4)	13 (41.9)
Weeks_injury-clinic entry_ M (SD)	6.2 (3.0)	5.1 (3.3)	6.3 (3.5)	6.4 (3.3)	5.6 (3.2)
Baseline RPQ M (SD)	42.5 (10.8) CI = 38.4, 46.6	43.8 (6.7) CI = 39.0, 48.6	45.0 (8.1) CI = 40.9, 49.0	40.9 (8.2)^†^ CI = 37.5, 44.4	46.2 (8.6)^†^ CI = 43.0, 49.4
Weeks_injury-follow-up_M (SD)	35.0 (3.6)	29.2 (5.2)	35.7 (4.8)	33.8 (5.4)	–
Follow-up RPQ M (SD)	24.2 (10.5) CI = 15.6, 32.8	31.5 (11.0) CI = 24.0, 39.0	34.1 (9.5) CI = 28.0, 40.2	31.0 (10.6) CI = 26.7, 35.27	–
RPQ change M (SD)	9.3 (6.5) CI = 0.9, 17.8	11.8 (12.5) CI = 4.5, 19.0	9.5 (9.2) CI = 3.0, 16.0	10.2 (9.5)^†^ CI = 6.2, 14.2	–
WHODAS M (SD)	20.8 (9.1) CI = 13.6, 28.1	27.3 (9.2) CI = 21.0, 33.5	32.1 (8.0) CI = 26.9, 37.2	28.0 (9.4) CI = 24.2, 31.8	–

† Significant at p < 0.05; CI = 95%.

RPQ: Rivermead Post Concussion Symptoms Questionnaire; WHODAS: World Health Organization Disability Assessment Schedule 2.0 (12-item interview).

Symptom burden decreased over time (t[23] = 5.2, p < 0.01), although remained elevated at follow-up (RPQ>24) across intervention conditions (*M* = 31.0, SD = 10.6). The degree of symptom reduction was comparable across intervention conditions (F[2, 21] = 0.15, p = 0.87). Once differences in baseline symptom burden were accounted for, there was no significant difference in disability (WHODAS) between intervention conditions (F[2, 20] = 1.40, p = 0.27).

At follow-up, 73% (8/11) of patients who completed the coping skills intervention reported that they were at least ‘somewhat satisfied’ with the group and 91% (10/11) would likely or very likely recommend it to a friend with mTBI.

## Discussion

We conducted a secondary analysis of a clinical registry to evaluate the effectiveness of a brief coping skills group intervention for adults with high symptom burden following mTBI. We compared self-reported outcomes between participants who completed, declined/discontinued or were not offered the coping skills group because of staffing unavailability. Symptom burden decreased significantly over time; however, the rate of improvement was comparable across intervention conditions. Thus, our results do not support the effectiveness of this coping skills intervention beyond treatment as usual (e.g., psychoeducation). Nevertheless, patients reported moderate to high satisfaction with and perceived credibility of the intervention.

While it is possible that the coping skills intervention provided no clinical benefit beyond an in-person education session, there are several alternative reasons that may underlie our found null effects. Given that the small group differences favored those receiving no additional intervention, it is unlikely that one of these reasons was insufficient statistical power. Rather, one possible reason is that the intervention was not sufficiently potent. It was designed to be very brief for feasibility purposes, but this may have come at a cost to effectiveness. It is possible that a larger or more concentrated ‘dose’ of therapy may be required, especially for highly symptomatic patients at risk for poor outcomes. Indeed, a three-session group is a very low dose for typical CBT trials [13,14]. Expanding on the content over a greater number of sessions or supplementing or replacing the group sessions with individual support may increase intervention effectiveness. Our use of a group format may help reconcile differences with prior studies that found CBT to be effective following mTBI, using individual therapy [5,15]. Potential benefits of group therapy (e.g., symptom normalization, enhanced motivation, modeling adaptive coping) [16] may be outweighed by disadvantages (e.g., symptom contagion or other iatrogenic effects) in the context of mTBI.

Additionally, the outcome measures used in this study may have been too indirect and, therefore, insensitive to potential intervention effects. Although the direct intervention targets were coping, self-efficacy and modified illness perceptions, the outcome measures narrowly assessed the number and intensity of symptoms. Another potential reason for the lack of apparent intervention effect is the patient selection method. Patients were invited to the group on the basis of being highly symptomatic; however, it may be more productive to offer CBT to patients who demonstrate maladaptive coping instead (i.e., who display the specific psychological risk factors that CBT is designed to modify). Finally, patients who declined or were not offered the coping skills intervention may have been more likely to receive additional private treatment.

### Study limitations

Several study limitations warrant mentioning. First, participants were not randomly assigned to treatment conditions. Some were not offered the intervention of interest based on inadequate staffing availability at the time they presented to clinic, creating a comparison (no additional intervention control) group. The decision to offer versus not offer the coping skills group was independent of patient characteristics and so can be considered pseudo-random. Nevertheless, the intervention groups appeared generally comparable on measured demographic and injury characteristics, as well as baseline symptom severity. Another major limitation was high (55%) loss to follow-up, particularly of those with highest initial symptom burden. This degree of missing follow-up data and the likelihood of nonrandom mechanisms contributing to missingness may be an important source of bias in this study, further tempering conclusions about treatment (in)effectiveness. Additionally, we relied on the referring physician to diagnose mTBI. We analyzed data from a clinical program that did not impose particular diagnostic criteria on referrers or provide routine diagnostic verification. Finally, because the intervention was delivered in a clinical context, the sessions were not audio- or video-recorded, and so assessment of therapist fidelity to the treatment manual was not possible.

Future research may benefit from true randomization and more purposeful pre-post assessment design using measures specifically matched to intervention content or proposed mechanisms of therapeutic action. Last, the exploration of longer group formats or supplementation with individual therapy may prove fruitful.

## Conclusion

In this nonrandomized trial, treatment-seeking patients with high symptom burden after mTBI who completed a brief CBT-based coping skills group intervention perceived the group to be satisfactory and credible, but their symptom recovery trajectory and disability outcomes were comparable to patients who did not complete the group. More intensive intervention may be required for this patient subpopulation.

## Future perspective

Psychological factors are increasingly recognized as potent modifiers of recovery from mTBI. Over the next decade, we expect that evidence-based theoretical models will advance to specify which psychological factors are most important and how they (mechanistically) lead to symptom chronicity. We will be able to apply principles of precision medicine to better identify patients with particular psychological risk factors for poor mTBI outcome and target therapy to those risk factors. The optimal timing, dose and format of psychologically informed treatment will also be better established.

**Summary points**

**Rationale**

- Psychosocial factors are important perpetuating factors of prolonged symptoms following mild traumatic brain injury (mTBI).
- Early psychotherapeutic intervention may be beneficial to those with mTBI, particularly those with severe symptoms.

**Intervention**

- A three-session coping skills group intervention was designed to help patients develop a graduated return to activity plan and learn how to use cognitive reframing and relaxation techniques.

**Results**

- At follow-up, patients who completed the coping skills intervention reported no more symptom improvement than patients who had received treatment as usual.
- The majority of patients who completed the coping skills intervention reported that they were at least ‘somewhat satisfied’ with the group and would likely or very likely recommend it to a friend with mTBI.

**Recommendations**

- More intensive intervention (e.g., more sessions) may be necessary for this patient subpopulation.
- Future studies would benefit from true randomization and selection of outcome measures matched to the specific content of the intervention.
